# Biological Properties of *Boletus edulis* Extract on Caco-2 Cells: Antioxidant, Anticancer, and Anti-Inflammatory Effects

**DOI:** 10.3390/antiox13080908

**Published:** 2024-07-27

**Authors:** Javier Quero, Mónica Paesa, Carmen Morales, Gracia Mendoza, Jesús Osada, José António Teixeira, Pedro Ferreira-Santos, María Jesús Rodríguez-Yoldi

**Affiliations:** 1 Department of Pharmacology and Physiology, Forensic and Legal Medicine, Veterinary Faculty, University of Zaragoza, 50013 Zaragoza, Spain; javierquero94@gmail.com (J.Q.); 776076@unizar.es (C.M.); gmendoza@iisaragon.es (G.M.); 2 Department of Chemical Engineering, University of Zaragoza, Campus Río Ebro-Edificio I+D, C/Poeta Mariano Esquillor S/N, 50018 Zaragoza, Spain; 683886@unizar.es; 3 Institute of Nanoscience and Materials of Aragon (INMA), CSIC-University of Zaragoza, 50009 Zaragoza, Spain; 4 Aragon Health Research Institute (IIS Aragon), 50009 Zaragoza, Spain; 5 Department of Biochemistry and Molecular Cell Biology, Veterinary Faculty, University of Zaragoza, 50009 Zaragoza, Spain; josada@unizar.es; 6 CIBERobn, ISCIII, IIS Aragón, IA2, 50009 Zaragoza, Spain; 7 CEB-Centre of Biological Engineering, University of Minho, Campus de Gualtar, 4710-057 Braga, Portugal; jateixeira@deb.uminho.pt; 8 LABBELS—Associate Laboratory, Braga/Guimarães, 4710-057 Braga, Portugal; 9 Department of Chemical Engineering, Faculty of Science, University of Vigo, 32004 Ourense, Spain; 10 IAA—Instituto de Agroecoloxía e Alimentación, University of Vigo (Campus Auga), As Lagoas, 32004 Ourense, Spain

**Keywords:** *Boletus edulis*, Caco-2 cells, antioxidant, anti-inflammatory, anti-cancer

## Abstract

*Boletus edulis* (BE) is a mushroom well known for its taste, nutritional value, and medicinal properties. The objective of this work was to study the biological effects of BE extracts on human colon carcinoma cells (Caco-2), evaluating parameters related to oxidative stress and inflammation. In this study, a hydroethanolic extract of BE was obtained by ohmic heating green technology. The obtained BE extracts are mainly composed of sugars (mainly trehalose), phenolic compounds (taxifolin, rutin, and ellagic acid), and minerals (K, P, Mg, Na, Ca, Zn, Se, etc.). The results showed that BE extracts were able to reduce cancer cell proliferation by the induction of cell cycle arrest at the G0/G1 stage, as well as cell death by autophagy and apoptosis, the alteration of mitochondrial membrane potential, and caspase-3 activation. The extracts modified the redox balance of the cell by increasing the ROS levels associated with a decrease in the thioredoxin reductase activity. Similarly, BE extracts attenuated Caco-2 inflammation by reducing both *iNOS* and *COX-2* mRNA expression and COX-2 protein expression. In addition, BE extracts protected the intestine from the oxidative stress induced by H_2_O_2_. Therefore, this study provides information on the potential use of BE bioactive compounds as anticancer therapeutic agents and as functional ingredients to prevent oxidative stress in the intestinal barrier.

## 1. Introduction

Colorectal cancer (CRC) is one of the principal causes of cancer death [1]. The occurrence of this disease is increasing (one million new cases are detected every year), which means that it is still necessary to look for natural sources with preventive properties (such as personalized foods, supplements, etc.) and more effective alternatives for its treatment. In this sense, derivatives of natural compounds can be used as cancer prevention agents due to their low toxicity [2]. Therefore, preventing CRC by changing unhealthy dietary habits and consuming foods with bioactive molecules, especially with antioxidant and anticancer actions, seems to be the most effective strategy to decrease CRC incidence [3]. Edible mushrooms, particularly Basidiomycetes, are important components of our daily diet due to their nutritional value and benefits for human health. Mushroom-derived preparations are used in modern clinical practice in Asian countries [4]. These preparations can contain valuable compounds such as polysaccharides, proteins, lipopolysaccharides, glycoproteins, lectins, polysaccharide–protein complexes, and metabolites like phenolic compounds, lactones, and terpenoids with different actions and activities. Interestingly, mushrooms also contain enzymes with important actions in the body’s metabolism, like laccase, superoxide dismutase, glucose oxidase, and peroxidase [5].

Mushroom compounds have antitumor, immunomodulatory, antioxidant, antiviral, antimicrobial, antiparasitic, hepatoprotective, antidiabetic, and antihypercholesterolemic effects [6]. Given that some edible mushrooms contain many chemicals that are effective in the treatment of cancers, they would be suitable candidates for this approach [7,8]. It has long been documented that the natural molecules of Basidiomycetes, such as polysaccharides, are effective against cancer without toxic effects in vivo [9,10]. Studies have established that these polysaccharides show anticancer action by activating the host immune system and are considered modifiers of biological responses [11,12]. However, various glucans have also been shown to exert direct growth-inhibitory properties and induce cell death in various cancer cells [11,13,14].

*Boletus edulis* (BE) is a popular edible mushroom, both in terms of demand and its frequent use in food. Despite its widespread popularity, little is known about its effects on human health and its medicinal uses. However, several reports have demonstrated the possibility of using some molecules like polysaccharides and proteins recovered from BE in cancer treatment. In fact, in 1957, Lucas et al. [15] used extracts isolated from BE fruiting bodies in mice models of cancer and obtained promising results in the treatment of tumors. In 1973, Ohtsuka et al. [16] also showed the anticancer action of polysaccharide fractions from BE against Sarcoma 180 and Ehrlich solid tumors in mice. Other studies have shown that BE has an antiproliferative effect in colon cancer cells (LS180) [17]. In this sense, several research groups [17,18,19] have extensively studied the mechanism of action of BE extract in two colon cancer cells, LS180 and HT-29. The results showed that the BE extract is a potent p53-dependent apoptosis inducer in human colon cancer cell lines with small differences between these cell lines. In addition, the infrared spectra (FTIR) of the BE fraction confirmed it is composed of ribonucleic acid. Similarly, Feng et al. revealed the antiproliferative action of sesquiterpenoids from BE in the human colon cancer cell line SW480 using an MTT assay [20]. The same effect was observed by Bovi et al. [21], which was induced by lectin in the human colon cancer cells Caco-2 and HT-29. However, there is still a lack of knowledge about the chemical structure of its molecules and the mechanism responsible for its anticancer action. This study aimed to investigate the effect and mechanism of action of a hydroethanolic extract of *B. edulis* obtained by ohmic heating on cell proliferation, redox balance, and inflammation in human colon carcinoma Caco-2 cells. Furthermore, the overall objective was to understand the protective effect of these BE extracts on the intestinal barrier.

## 2. Materials and Methods

### 2.1. Boletus edulis Characterization

Wild-growing BE fruiting bodies were collected in a mixed pine/oak forest in Albarracin (Teruel, Spain). The collected BE fruiting bodies were similar in size (with fresh weight of around 70 g) and had not reached full maturity; i.e., they were in the young/white stage.

Samples were lyophilized for 4 consecutive days to a final humidity of 4%, which is important to ensure spore inactivation and prevent contamination/proliferation of microorganisms. The mushrooms were then ground into powder. This was analyzed for its chemical composition (moisture, proteins, fat, carbohydrates, and ash) according to the official protocols of the National Renewable Energy Laboratory (NREL). The crude protein content (N × 4.45) of the BE was assessed by the macro-Kjeldahl method; the fat content of BE was determined gravimetrically after petroleum ether extraction using a Soxhlet extractor; and the ash content was calculated after incineration at 575 °C to constant weight. Total extractives were determined by Soxhlet exhaustive extraction using water and ethanol. Total carbohydrates were calculated by difference. Minerals were quantified by inductively coupled plasma atomic emission spectrometry after BE digestion with nitric acid.

Energy of BE was considered using the resulting equation as follows:

$$Energy\;\left(kcal\right)=4\times (protein+carbohydrate)+9\times fat$$

### 2.2. Extraction of Boletus edulis Bioactive Compounds

For the extractions, different conditions were tested to obtain a more effective recovery of bioactive compounds. The conditions used in this study were as follows: 15 g of dry BE fruiting bodies powder was mixed with 150 mL of 70% hydroethanolic solution (2.1 mS/cm electrical conductivity after 5 min of stirring) for 30 min at 55 °C, with stirring (150 rpm). The obtained BE extracts (BE) were centrifuged for 10 min at 8000× *g* at 10 °C (Fiberlite™ F14–6x250LE Fixed Angle Rotor, Thermo Scientific™, Waltham, MA, USA), and the supernatant was filtered (10 µm) and stored at −20 °C until further analysis. Extractions were performed in an ohmic heating (OH) glass reactor (with a total capacity of 250 mL) with two electrodes (with a distance of 5 cm between them) and worked at an electric field of 13 V/cm at 25 kHz. An oscilloscope (ScopeMeter R 125/S, Fluke, Washington, DC, USA) was used to measure electrical frequency, voltage, and current intensity.

### 2.3. Chemical Composition Extract

#### 2.3.1. Total Phenolic and Flavonoid Content

Total phenolic content (TPC) was analyzed using Folin–Ciocalteu colorimetric assay adapted for microplates [22], with 10 µL of BE extract. Gallic acid was used as a standard compound (50–1500 mg/L, R^2^ = 0.997), and the data are expressed as milligrams of gallic acid equivalents (GAE) per gram of dry *B. edulis* extract (mg GAE/g BE).

The total flavonoid content (TFC) was measured using aluminum chloride colorimetric assay as described by Ferreira-Santos et al. [23]. Catechin (4–400 mg/L, R^2^ = 0.998) was used for method calibration, and the obtained data are expressed as mg of catechin equivalents (CE) per g of dry *B. edulis* extract (mg CE/g BE).

#### 2.3.2. Phenolic Compound Identification and Quantification

The analysis of phenolic compounds in BE was carried out as previously reported by Ferreira-Santos et al. [22], using the injection volume of 5 μL. The separation was performed with a C18 column from Waters (Milford, MA, USA) using water with formic acid (0.1%) and acetonitrile as elution solvent with a flow of 0.4 mL/min (at a constant temperature of 40 °C). A liquid chromatograph equipped with a diode array detector (DAD) (Shimadzu, SPD-M20A, Columbia, MA, USA) was used. Calibration curves were performed using concentrations between 2.5 and 250 mg/mL for each compound, and the coefficient of linear correlation (R^2^) was greater than 0.99 for all curves. Specific wavelengths (250–370 nm) and retention times were used to quantify and identify each compound, as previously validated (see Supplementary Table S1).

#### 2.3.3. Sugar Identification and Quantification

The monosaccharide composition of the BE extract was examined by HPLC using an Agilent 1200 series instrument (Palo Alto, Santa Clara, CA, USA) coupled to an index refraction detector (at 35 °C). Five microliters of BE extract (after removal of ethanol and volume adjustment with water) were injected into an Organic Acid H+ column (Phenomenex, Torrance, CA, USA) using 3 mM H_2_SO_4_ as mobile phase at a flow rate of 0.6 mL/min (at a constant temperature of 60 °C). Sugar compounds were used as standards for identification and quantification (trehalose, rhamnose, mannitol, glucose, and fructose).

#### 2.3.4. Antioxidant Activity of the Extract

The antioxidant capacity of BE was evaluated using three different assays according to the following methods reported by Ferreira-Santos et al. [22]: ferric reducing antioxidant power (FRAP), radical cation decolorization (ABTS), and free radical scavenging (DPPH). The FRAP method was performed, and the results were compared with those of ferrous sulfate solution (800–100 μM, R^2^ = 0.996) and are expressed in μmol Fe^2+^/g BE extract. The DPPH and ABTS results are reported in 6-hydroxy-2,5,7,8-tetramethylchroman-2-carboxylic acid (Trolox) equivalents (μmol TE/g BE extract) compared to the respective calibration curves for DPPH (with 15–400 µM, R^2^ = 0.998) and for ABTS (with 15–800 µM, R^2^ = 0.997).

### 2.4. Cell Culture and Viability

The human Caco-2 cell line (clone TC7) was generously provided by Dr. Edith Brot-Laroche (Université Pierre et Marie Curie-Paris 6, France) and kept in a humidified atmosphere of 5% CO_2_ at 37 °C. Cells were cultured in Dulbecco’s modified Eagle’s medium (DMEM) (Gibco Invitrogen, Paisley, UK) complemented with 20% fetal bovine serum (FBS), 1% nonessential amino acids, 1% penicillin (1000 U/mL), 1% streptomycin (1000 μg/mL), and 1% amphotericin (250 U/mL) (DMEM^+^). Cells were cultured in flasks (25 cm^2^) at a 5 × 10^5^ cells/cm^2^ density, and DMEM^+^ was substituted every 2 days. BE treatments were added 24 h after plating for assays in undifferentiated Caco-2 cells [24] and 15 days after plating on differentiated Caco-2 cells. Cell confluence (80%) was observed by microscopy.

BE extracts were prepared in DMEM^+^ to a concentration of 1.5 mg/mL, taking into account preliminary tests and previous studies performed in our laboratory with other natural extracts. For the determination of cytotoxicity, cells were seeded in 96-well plates at a density of 4 × 10^3^ cells/well for 24 h. After, cells were incubated with the different concentrations of BE extracts for 24, 48, or 72 h. The antiproliferative effect was determined using a resazurin cell viability fluorometric assay as previously described [25]. The results are represented in relation to the percentage of the control cells. The value of the extract concentration that reduces cell proliferation or viability by half (IC_50_) is selected for additional analysis to clarify its mechanism of action on Caco-2 cells.

### 2.5. Cell Cycle Analysis and DNA Content

To study the action of BE extract on cell cycle and DNA content, Caco-2 cells were treated with the IC_50_ concentration for 48 h. After, the cells were fixed in ice-cold 70% ethanol and maintained at 4 °C for 24 h. Additionally, the cells were centrifuged at 2500 rpm for 5 min and rehydrated in PBS, and 50 μg/mL propidium iodide (PI) was used for staining with RNase A (100 μg/mL). Treated cells were studied for DNA content using a BD PCSarray [26].

### 2.6. Cell Apoptosis Evaluation

To understand the type of cell death promoted by the BE extracts, cells were cultured with the extracts (at the IC_50_ concentration for 48 h), at 5 × 10^5^ cells/cm^2^ in 25 cm^2^ flasks. After, the cells were stained with Annexin V-FITC and propidium iodide as previously described [26] and analyzed by flow cytometry as previously reported [25]. The untreated cells (negative control) were prepared to define the basal level of apoptotic and necrotic or dead cells.

### 2.7. Determination of Mitochondrial Membrane Potential and Caspase-3

To determine mitochondrial potential and caspase-3 activity, a method previously described by our research group was used [25]. For that, cells were cultured in 25 cm^2^ flasks at 5 × 10^5^ cells/cm^2^ and then exposed to BE extracts (at the IC_50_ concentration) for 48 h. Control cells were incubated only with DMEM^+^.

### 2.8. Autophagy Determination

Caco-2 cells were placed in 96-well microplates at 4 × 10^3^ cells/well and treated with BE extracts for 24 h. Autophagosome formation was determined following instructions of the Autophagy Assay Kit (MAK138-1KT Sigma-Aldrich, St. Louis, MO, USA). Fluorescence intensity was measured at λex = 360/λem = 520 nm with FLUOstar Omega plate reader, and the obtained fluorescence intensity values are measured as a reflection of the total autophagosome formation and thus autophagy.

### 2.9. Determination of Intracellular Levels of Reactive Oxygen Species (ROS)

The intracellular level of ROS was measured by dichlorofluorescein assay as previously described [25]. Cells were placed in 96-well plates at 4 × 10^3^ cells/well and treated with BE extracts for 24 and 48 h. ROS levels were reported as a percentage of fluorescence compared to control. The fluorescence intensity values obtained are considered to reflect the total level of intracellular ROS.

### 2.10. Determination of Thioredoxin Activity

A kit (Sigma^®^ CS0170) was used to study the effect of BE extracts on the enzyme thioredoxin reductase in vitro. Slight modifications were made to the protocol to adapt it to the interaction with the recombinant human enzyme TrxR1 (Sigma^®^ SRP6081), specifically the cytosolic isoform. The assay was carried out in 96-well microplate containing the assay buffer (500 mM PBS, pH 7.0, 50 mM EDTA) and a reaction buffer consisting of the above buffer together with NADPH. The extracts were incubated at the IC_50_ concentration with 2 μL of the enzyme (0.5 mg/mL) at 25 °C. Each well was filled with 180 μL reaction buffer. Finally, 6 μL of 100 mM 5,5′-dithiobis-(2-nitrobenzoic acid) (Ellman’s Reagent, DTNB) (in pure DMSO) was added as an enzyme substrate to start the reaction. To determine the activity, the absorbance was measured at 412 nm in cycles of 30 s for 22 min using a FLUOstar Omega multiplate reader (BMG Labtech, Ortenberg, Germany). The data are reported as the percentage of fluorescence relative to the control.

### 2.11. mRNA Expression Levels of iNOS and COX-2

After treating the Caco-2 cells with BE extracts at the IC_50_ concentration for 48 h, the cells were harvested to assess the gene expression associated with inflammation using quantitative reverse transcription PCR (RT-qPCR). The BE extracts were administered to the cells either 24 h after seeding for undifferentiated Caco-2 cells or after 15 days for differentiated cells. PBS was used to lyse the cells, and total RNA was isolated from the collected samples using the TRIzol extraction technique. The extracted RNA concentration and purity were measured using NanoDrop 2000 spectrophotometer (Thermo Fisher Scientific, Waltham, MA, USA). Reverse transcription of RNA to complementary DNA (cDNA) was accomplished using the PrimeScriptTM RT Master Mix (Takara Bio, Shiga, Japan) from 500 ng of total RNA. Amplification was performed using the QuantStudioTM 5 Real-Time PCR Instrument (Applied Biosystems, Waltham, MA, USA) at 95 °C for 30 s, followed by 40 cycles of 95 °C for 5 s and 60 °C for 30 s. The reaction was carried out in a total volume of 20 μL, mixing 2 μL diluted cDNA, 2 μL primers, 10 μL MasterMix (Takara Bio, Shiga, Japan), 5.64 μL sterile water, and 0.36 μL of ROX Reference Dye II (Takara Bio, Shiga, Japan). Human *NOS2* (Hs.PT.58.14740388) and *COX-2* (Hs.PT.58.77266) target probes (Integrated DNA Technologies, Park Coralville, IA, USA) were used to evaluate gene expression, and the level of the target mRNA was normalized to the level of *GADPH* (Hs.PT.39a.22214836). The 2^−ΔΔCT^ technique was employed to calculate the relative expression levels of the target genes. Three replicates of each gene from three separate experiments were performed.

### 2.12. COX-2 Immunofluorescence

Caco-2 cells were seeded on sterile coverslips at a rate of 3 × 10^5^ cells/cm^2^ and treated or not with BE extracts at the IC_50_ concentration for 48 h. After treatment, the cells were fixed for 15 min at room temperature in 4% paraformaldehyde (Alfa Aesar, Karlsruhe, Germany) after being rinsed with PBS. After fixation, the cells were successively cleaned with 0.1% saponin in BSA–PBS solution and 1% BSA in PBS. After that, the samples were incubated with a primary antibody against COX-2 (1:50; Abcam, Cambridge, UK) for an entire night at 4 °C. Subsequently, the cells were treated for one hour at room temperature with Alexa Fluor 488 goat anti-rabbit IgG antibody (1:250; Molecular Probes, Eugene, OR, USA). Phalloidin 546 (1:50; Molecular Probes, USA) was used to label f-actin in a PBS–BSA-saponin solution. Following that, 1% BSA in PBS and distilled water were used to wash the cells. Finally, coverslips were stained with 2 μg/mL of DAPI (Molecular Probes, USA) at 25 °C for 15 min before being mounted on glass slides with Mowiol mounting medium (Thermo Fisher, USA). Laser scanning confocal microscope (Leica TCS SP2, Wetzlar, Germany) was used to analyze the samples. The experiment was executed in triplicate.

### 2.13. Statistical Analyses

The results are presented as mean ± SD. A one-way analysis of variance was used to compare the means (ANOVA). We used the Bonferroni multiple comparison test to compare differences that were statistically significant at *p* < 0.05. Software from GraphPad, San Diego, CA, USA—GraphPad Prism version 5.02—was used for statistical analysis and graphing. Every test was run with at least three replicas.

## 3. Results and Discussion

Natural chemical substances from living organisms such as animals, plants, and microorganisms have significant anti-cancer potential. Among these are edible and medicinal mushrooms with important therapeutic potential. Mushrooms contain important nutrients and biologically active molecules (such as carbohydrates, proteins, polyphenols, minerals, and vitamins) [27] and have a variety of medicinal properties such as anti-tumor, antioxidant, antiviral, and others [28]. Therefore, the aim of this work was to evaluate the bioactive compounds present in BE extracts and their application in the treatment of colon cancer, focusing on their possible antiproliferative, antioxidant, and anti-inflammatory effects.

### 3.1. Approximate Composition of BE

The chemical composition of the BE used in this study is presented in Table 1. The results demonstrated that the major fractions of this mushroom’s composition were carbohydrates, representing 58.1%. The ethanol and water-soluble extractives represented 43.5% and 36.8% of the BE composition, respectively. The protein content quantified by the total nitrogen concentration of the BE was 19.7%. Inorganic matter (ash) represented 5.4% of the total composition, and the major minerals, as determined by plasma atomic emission spectrometry, were potassium, phosphorus, magnesium, sodium, calcium, zinc, aluminum, iron, and selenium (Table 2). Other constituents, such as the lipid fraction (fat), represented only 2.4% of the total BE content.

Several studies have reported the composition of BE mushrooms from different regions of the world [29,30]. In general, the composition of the BE used in this work was similar to that reported in these studies, assuming that the small differences were due to the growing conditions of the mushroom, such as soil composition, environmental conditions (temperature, humidity, etc.), and geographical location.

Overall, the characteristics of these mushrooms prove to be a good source of several important dietary components.

### 3.2. Chemical Composition of BE Extracts

The results of the TPC and TFC of the hydroethanolic extracts of BE are presented in Table 3. The extraction method used (13 V/cm OH with 70% ethanol for 30 min at 55 °C) led to an extraction yield of about 32%. The extracts obtained showed a TPC concentration of 79 ± 4 mg GAE/g extract and a TFC of 7.9 ± 0.9 mg CE/g extract. These BE extracts have interesting antioxidant activity, showing antioxidant reducing power values of 103 ± 6 µmol Fe^2+^/g extract for FRAP as well as radical scavenging effects of 17 ± 0.1 µmol TE/g extract for DPPH and 52 ± 2.5 µmol TE/g extract for ABTS.

In addition, individual phenolic compounds of BE extracts were identified and quantified by chromatography, and the results are shown in Table 3. A total of nine phenolic compounds were identified: four flavonoids and five phenolic acids. The most representative phenolics were ellagic acid (532 µg/g extract), rutin (465 µg/g extract), and taxifolin (259 µg/g extract), with the others found in concentrations between 173 and 53 µg/g extract. Large discrepancies have been observed when comparing the phenolic content of BE extracts [31,32]. These variations are most likely due to the extraction techniques used, the origin of the BE extracts, and the possibility that their phenolic contents may be expressed differently.

Several epidemiological studies have demonstrated a correlation between adherence to a Mediterranean-based diet (which includes a high intake of cereals, fruits, vegetables, mushrooms, etc.) and a reduced risk of developing diseases related to oxidative stress, including cancer, inflammation, osteoarthritis, cardiovascular diseases, diabetes, and others [25,33,34,35,36]. These protective effects may be attributed to the antioxidant and anti-inflammatory capacities of some phenolic compounds [37,38,39,40]. These are secondary metabolites produced by plants, algae, or fungi that represent the largest group of non-energy-yielding compounds (more than 8000 phenolic molecules have been identified). These include phenolic acids (hydroxycinnamic and hydroxybenzoic acids), coumarins, flavonoids (e.g., flavones, flavanones, flavonols, anthocyanins, etc.), and non-flavonoids (lignans, tannins, and stilbenes) [41,42,43].

Flavonoids are notable for their antioxidant properties, while their pharmacological actions are diverse and encompass anticancer, cardioprotective, neuroprotective, anti-inflammatory, anti-infectious, and other effects. A substantial body of research has investigated the potential association between flavonoid intake and cancer prevention. There is evidence indicating an inverse association, particularly of quercetin, with the risk of cancer [44]. Furthermore, flavonoids have been investigated in the context of gastrointestinal cancers. A meta-analysis has demonstrated that the consumption of various subclasses of flavonoids, including flavonols, flavones, and anthocyanins, may potentially reduce the risk of CRC. Notably, quercetin and apigenin have been demonstrated to reduce the risk of colon and rectal cancer, respectively [45]. Furthermore, the consumption of soy isoflavones, epicatechin, and procyanidins has been significantly associated with a reduced risk of colorectal [46] and gastrointestinal cancers [47]. However, it has been seen that these compounds when administered alone at high doses can produce side effects and that is why their administration in capsules or with other anticancer drugs is being studied [48]. Moreover, the incubation of Caco-2 cells with quercetin showed a reduction in cell viability and proliferation [49]. Likewise, Table 3 shows that ellagic acid is found in a large proportion. This phenolic acid shows synergistic effects in chemotherapy for the treatment of cancer and exerts prodigious potential in reducing the side effects of current therapy due to its antioxidant and anti-inflammatory capacities [50].

Conversely, the mushroom’s polysaccharides have demonstrated intriguing bioactive properties, including antioxidant and anticancer effects, contingent on their monomeric composition [51]. The sugar compositions of the BE extract are presented in Table 4. The total sugar concentration was reported to be 437.5 mg/g of extract. The extract was found to contain trehalose (341 mg/g), followed by rhamnose, mannitol, glucose, and fructose, which were identified as the primary sugars present.

Trehalose is a naturally non-reducing disaccharide, composed of glucose molecules. Glucose molecules released by trehalose can be actively absorbed and processed by intestinal mucosal cells through the glucose transporter (SGLT1) [52]. The human digestive tract produces trehalases, enzymes that metabolize trehalose. However, trehalose can also be metabolized by trehalases produced by microbes [53]. Despite the substantial increase in human consumption and utilization of trehalose, no adverse effects have been documented, with the exception of rare instances of malabsorption resulting from trehalase deficiency [54]. Animals do not synthesize trehalose, yet the beneficial properties of this sugar have been confirmed in several illnesses, including cancer, inflammatory phenomena, and other oxidative-stress-associated diseases [55,56,57]. Consequently, trehalose has been demonstrated to exhibit anticancer activity in in vitro models of human hepatocellular carcinoma, CRC, and gastric cancer and in vivo models of acute lymphoblastic leukemia [56,58]. Furthermore, trehalose has the potential to suppress tumor growth and proliferation due to its ability to decrease inflammation in tumor tissue [59]. In a similar vein, Yu et al. [60] demonstrated that trehalose can suppress the production of inflammatory mediators in LPS-induced macrophages, including prostaglandin E2 and nitric oxide. Additionally, trehalose may serve as a viable therapeutic agent to activate autophagy in various conditions, including cancer [61]. It has been demonstrated that autophagy may be able to inhibit the development and propagation of tumors by lowering oxidative stress in cancerous cells, in which mitochondrial damage promotes the generation of reactive oxygen species [62]. The capacity of trehalose to safeguard a multitude of biological entities, including DNA, proteins, cells, and tissues, has presented opportunities in the pharmaceutical and biotechnology sectors for its use in the development of novel therapeutic agents [63]. Moreover, trehalose has been demonstrated to regulate the human intestinal microbiota, which is important for maintaining intestinal health and for the consequent regulation of cognitive function. This is achieved by regulating blood–brain barrier permeability, brain energy homeostasis, and synaptic transmission [64].

One’s diet contains a number of vital micronutrients, such as minerals, that are crucial for preserving and improving the body’s functionality [65]. Mushrooms are a valuable bioresource of trace elements, including copper, zinc, selenium, and iron, which are essential for numerous biochemical processes within an organism. The specific type of mushroom, its habitat, the stages of growth, and the precipitation in the environment all affect the amounts of trace elements that these species are able to store during their development.

In comparison to other mushrooms, BE exhibits distinctive nutritional and pharmacological characteristics [32,66]. It contains substantial quantities of essential macrominerals, including calcium, magnesium, phosphorus, potassium, and sodium, as well as microminerals such as selenium, zinc, and copper [67,68]. These results are presented in Table 2, which displays the raw material composition. Of particular interest is the extraction of these minerals using a water–ethanol solvent in combination with ohmic heating, as detailed in Table 5. The table shows that selenium, zinc, copper, and iron were present. Selenium is a vital component in maintaining optimal health and regulating physiological processes [69,70]. These include the function of several enzymes [71] and the promotion of body homeostasis [72]. The detrimental effects of both excessively high and low selenium levels on human health are elucidated. For instance, oxidative stress resulting from inadequate selenium levels can reduce the body’s selenoprotein content, including glutathione peroxidases and thioredoxin reductases. Nevertheless, excessive selenium can also result in oxidative stress by forming reactive oxygen species through the oxidation and cross-linking of protein thiol groups [72]. The recognition of selenium’s antioxidant, anti-inflammatory, and anticarcinogenic properties suggests that BE could be utilized as a novel selenium source in food supplementation or as a value-added constituent for the preparation of functional foods or nutraceuticals.

Several studies have demonstrated a correlation between dietary zinc intake and the risk of cancer [73]. Zinc’s anticancer actions are frequently associated with its antioxidant properties. Moreover, numerous studies have demonstrated the impact of zinc supplementation on a range of cellular processes, including transcription factors, cell proliferation and differentiation, DNA and RNA synthesis and repair, enzyme activation or inhibition, cellular signaling regulation, immune system function, and the stabilization of cell membranes and structure [73,74].

### 3.3. Antiproliferative Effect of BE Extracts on Caco-2 Cells

The BE extracts, which were composed mainly of disaccharides (trehalose), phenolic compounds (taxifolin, rutin, and ellagic acid), and minerals (K, P, Na, Mg, Ca, Zn, and Se), were subjected to an investigation into their effects on the proliferation of human colon cancer cells (Caco-2). The test was conducted with concentrations of the BE extract between 250 and 2000 µg/mL and incubation times of 24, 48, and 72 h.

**Figure 1:**
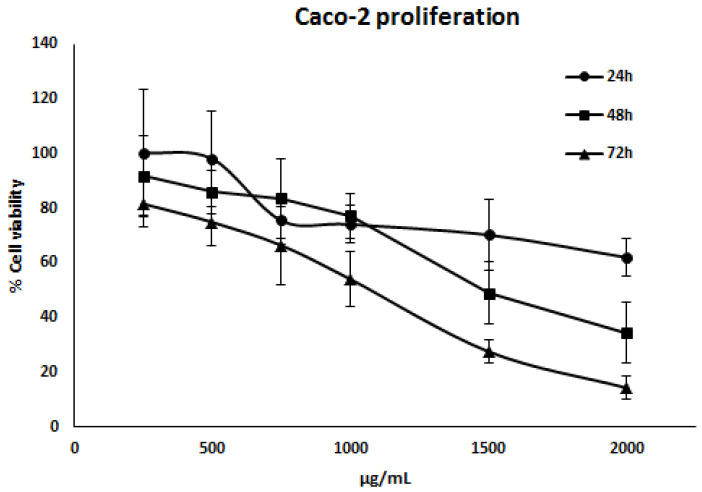
Cell viability of Caco-2 cells after incubation with *B. edulis* extracts (BE) at 250, 500, 750, 1000, 1500, and 2000 μg/mL for incubation times of 24, 48, and 72 h.

The results obtained, as illustrated in Figure 1, indicated that BE extracts exhibited a dose- and time-dependent reduction in the viability of Caco-2 cells. The data indicate that BE extracts displayed a significant antiproliferative effect, as evidenced by the IC_50_ values presented in Table 6. For subsequent experiments, the IC_50_ concentration corresponding to 72 h has been selected.

As previously stated, studies conducted in the food and pharmacology sectors have demonstrated that, in comparison to other mushrooms, BE exhibits a higher nutritional and pharmacological value due to its components [75]. Furthermore, BE plays an important role in the body and has anti-cancer, antioxidant, anti-diabetic, and anti-inflammatory activities [76]. Given the rising incidence of cancer, the remarkable anti-cancer potential of mushrooms has become a subject of interest to researchers and industries alike. In a published paper, Nowakowski and colleagues [77] summarize scientific data on the anti-cancer activity of mushroom extracts in different experimental models, including in silico, in vitro (cell lines), and in vivo (animal models as well as human case studies and randomized controlled trials). This review reported that mushrooms’ mechanisms in cancer therapy included the following: a reduction in the growth of cancer cells; an imbalanced percentage of cells in different phases of the cell cycle; an increase in autophagy and phagocytosis; an enhanced immune response; and the induction of cell apoptosis through the upregulation of pro-apoptotic factors and the downregulation of anti-apoptotic genes. Novakovic et al. [78] conducted a study to examine the phenolic content and bioactivity of water and hydroethanolic extracts of *B. edulis* collected from Serbia. The results demonstrated that both aqueous and ethanolic extracts exhibited noteworthy antioxidant activity, with the ethanolic BE extract exhibiting the most pronounced efficacy against the human breast MCF-7 cancer cell line (IC50 = 56 μg/mL). In a separate study, the authors reported that the water-solubilized polysaccharide–glycoprotein polymer (named BEP) from the fruiting bodies of *B. edulis* presents non-toxicity against normal colonic epithelial cells (CCD84 coTr). In contrast, the BEP exhibited a significant antiproliferative action, accompanied by cell cycle arrest in the G0/G1 phase in colon cancer cells (LS180) [79].

Zhang et al. isolated three distinct fractions of polysaccharides (BEPF30, BEPF60, and BEPF80) from the fruiting bodies of *B. edulis*. These polysaccharides have the potential to be utilized as natural compounds in functional foods to mitigate oxidative stress, as evidenced by their antioxidant properties [75]. BEL β-trefoil, obtained from the fruiting body of *B. edulis* [80], demonstrated the capacity to act on melanoma cells in a selective manner. BEL β-trefoil has been demonstrated to inhibit proliferation and migration by regulating the differential expression of RUNX2 in melanoma tumor cells [21,80]. Zhang et al. isolated 90 kDa nitrite reductase protein from the fruiting bodies of *B. edulis*. The peptide sequence of the protein is comparable to that of fungal nitrite reductase, which has been shown to prevent nitrite from being produced during pickling. In China, it has been used as a natural component in traditional Chinese medicine due to its beneficial effects on health [81].

### 3.4. Induction of Cell Death by BE Extracts

**Figure 2:**
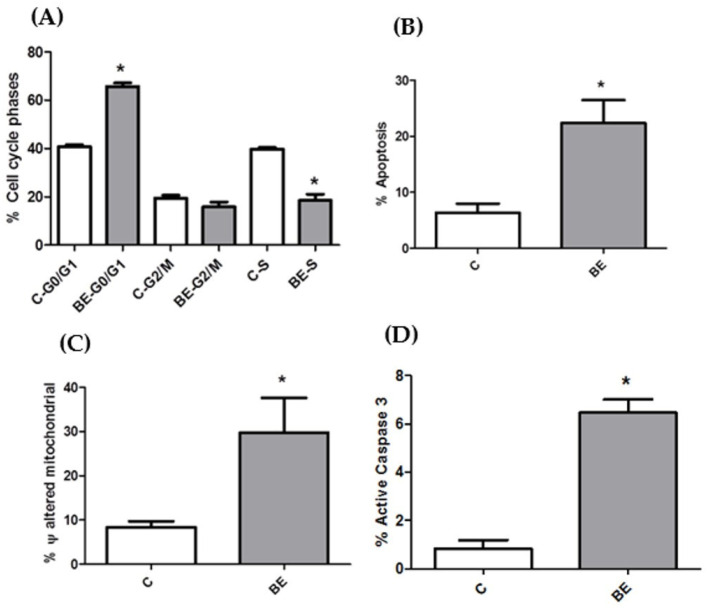
Caco-2 cell death studies by flow cytometry after incubation for 48 h with 1500 μg/mL (IC_50_) *B. edulis* extract (BE). (**A**) Percentage of cells in different phases of the cell cycle. (**B**) Percentage of cells in the apoptotic state. (**C**) Percentage of cells with disturbed mitochondrial membrane potential (ψ). (**D**) Percentage of cells with active caspase 3. * *p* < 0.05 compared to control (without treatment).

To elucidate the mechanism by which the BE extracts inhibited the proliferation of Caco-2 cells, the influence of these extracts on cell cycle stages was investigated by flow cytometry. The results demonstrated that these extracts induced the accumulation of Caco-2 cells in the G0/G1 cell cycle phase, concomitant with a reduction in cancer cells in the S phase (Figure 2A).

In a study by Lemieszek et al. [17], a water extract of BE, composed mainly of biopolymer polysaccharides and proteins, was tested on human colon adenocarcinoma cells (LS180). The results indicated that the extract inhibited cell proliferation, accompanied by cell cycle arrest in the G0/G1 phase. The authors observed that the growth inhibition was related to the regulation of the p16/cyclin D1/CDK4-6/pRb pathway, a critical stage in the progress of many human cancers, like colon cancer.

Other work has demonstrated the efficacy of an ethanolic extract of the medicinal mushroom *Antrodia cinnamomea* in inhibiting the proliferation of breast cancer cells (T47D) and inducing autophagy. This extract has been shown to arrest cells at the G1 stage of the cell cycle [82].

The observed changes in the cell cycle stages of the BE-treated Caco-2 cells prompted the next step in this study, which was to determine the type of cell death produced.

Apoptosis, or type I programmed cell death, can be initiated by a variety of signals in target cells. However, these signals ultimately converge at a single event: the release of cytochrome c into the cytosol due to damage to the mitochondrial membrane permeability, caspase activation, membrane blebbing, DNA fragmentation, and, finally, cell death [83]. Given that BE extract contains phenolic compounds and that our previous studies have demonstrated the ability of these compounds to induce apoptosis in cancer cells [36], we deemed it pertinent to investigate this specific form of cell death in the present work. The results of the apoptosis assay (Figure 2B) demonstrated that the BE induced apoptosis in the Caco-2 cells.

One of the initial steps in the apoptotic process is mitochondrial depolarization and caspase-3 activation. In light of our previous studies with natural extracts, which indicated mitochondrial dysfunction and the induction of intrinsic apoptosis in Caco-2 cells [36], the present work aimed to analyze the alteration of mitochondrial membrane potential and caspase-3 activity. The results presented in Figure 2C demonstrate that the BE extracts significantly alter the number of cells exhibiting mitochondrial potential changes, and caspase-3 is activated in the presence of 1500 µg/mL of BE extract over a 48 h period (Figure 2D). Consequently, these results corroborate the findings of apoptosis in colon cancer cells (Figure 2B) due to the effect of BE extracts, which are rich in phenolic compounds, trehalose, and essential microminerals, in modulating mitochondrial functions by inhibiting organelle enzymes or metabolic pathways.

A study conducted by Gibellini et al. [84] revealed that several natural compounds (quercetin, resveratrol, and curcumin) influence mitochondrial function by inhibiting transcription factors, regulating ROS production, and blocking enzymes or metabolic pathways. The aforementioned compounds demonstrated potent antioxidant actions, resulting in the release of cytochrome c from mitochondria and the upregulation of proapoptotic Bcl-2 family proteins, thereby exerting proapoptotic effects.

Mushrooms are a rich source of bioactive molecules, and their effects on cancer cells have been extensively studied. In particular, they have been shown to inhibit cancer cell proliferation and induce apoptosis, which could have important implications for cancer treatment. Some studies have demonstrated that a small RNA fraction extracted from BE has anticancer effects against colon cancer cells. These effects appear to be linked with the alteration of cell cycle arrest reflecting the signal transduction of the MAPK/Erk pathway and apoptosis induction [17,18,19].

Autophagy (type II programmed cell death) is a process involving the lysosomal turnover of proteins and organelles for the preservation of cellular homeostasis and the mitigation of cellular stress. A reduction or abnormality in autophagy impairs the degradation of damaged compounds or proteins in oxidative-stressed cells, which can lead to the development of aging and cancer. In cancer cells, autophagy suppresses tumorigenesis by the induction of cell death and reduction in cancer cell survival [62]. Consequently, basal autophagy is regarded as a contributing factor in the suppression of cancer.

**Figure 3:**
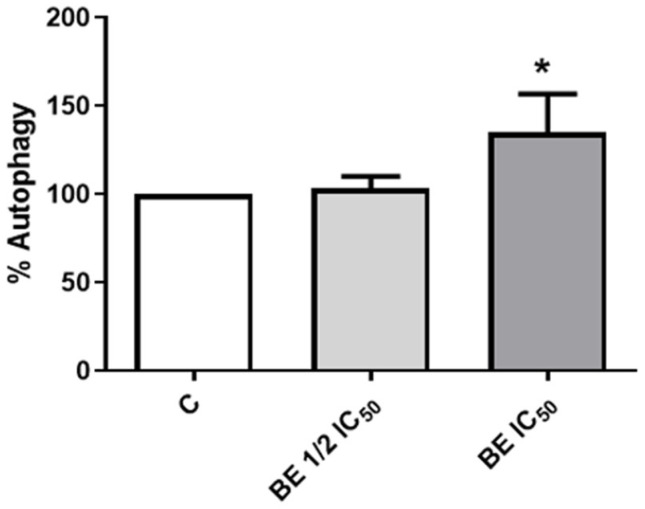
Autophagy induction. Measurement of changes in the formation of autophagosomes on Caco-2 cells 24 h after incubation with *B. edulis* extracts at 750 (1/2 IC_50_) or 1500 μg/mL (IC_50_). * *p* < 0.05 compared to control (without treatment).

In our research, we investigated the effects of autophagy cell death. Our findings, presented in Figure 3, indicate that BE extracts induce a moderate formation of autophagosomes, thereby inducing autophagy. Trehalose, a significant component of the BE extract, has been demonstrated to exert apoptotic and autophagic effects [85,86]. Moreover, numerous studies have verified that phenolic compounds, which are present in the BE extract, can effectively regulate autophagy in various types of cancer [87]. A number of authors have reported the action of different mushroom extracts (including *Antrodia salmonea*, *Antrodia cinnamonea*, *Phellinus linteus*, *Ganoderma lucidum*, and others) in inducing autophagy in cancer cells, thereby promoting anti-proliferative actions [82,88,89,90,91,92]. These findings indicate that the induction of cell death through autophagy by mushrooms may serve as an alternative to apoptosis, with therapeutic potential in cancer treatment. It is important to consider the potential of these natural extracts to provide a means of cancer cell death that enhances the effects of standard therapies when designing novel therapeutic strategies or as a coadjutant in actual chemotherapy.

### 3.5. Effect of BE Extracts on Redox Activity of Colon Cancer Cells

**Figure 4:**
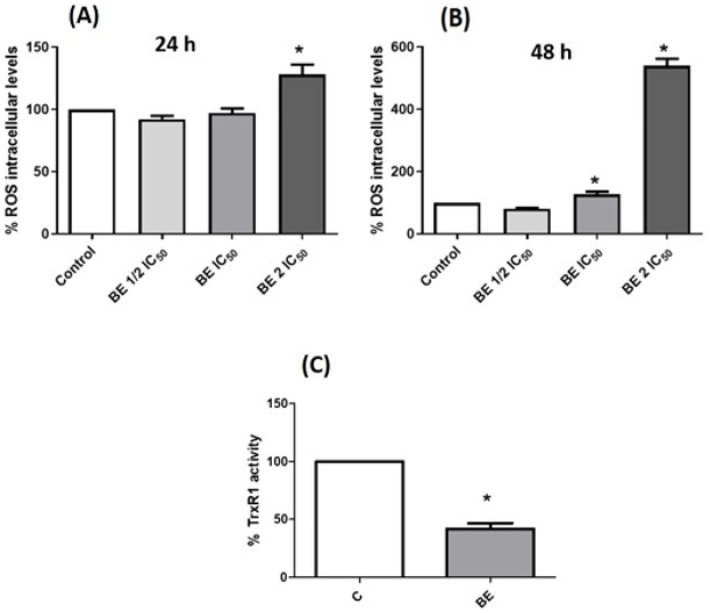
Redox activity on Caco-2 cells with 750 (1/2 IC_50_), 1500 (IC_50_), or 3000 (2 IC_50_) μg/mL *B. edulis* extract (BE). Measurements of the intracellular levels of ROS at 24 (**A**) and 48 h (**B**) of incubation time respectively. Determination of the reductase activity of recombinant TrxR1 after incubation for 48 h with BE extract at IC_50_ concentration (**C**). * *p* < 0.05 compared to control (without treatment).

It is postulated that diseases such as cancer are related to the formation of free radicals and their derived reactive products [93]. Cancer cells exhibit heightened metabolic activity, which results in elevated ROS levels and the subsequent activation of signaling pathways or mitochondrial dysfunction [94]. The results of the present study demonstrated that BE extracts, following a 24 h incubation period, exhibited a pro-oxidant effect at concentrations that were twice as high as the IC_50_ (labelled as 2 IC_50_ in Figure 4A). After 48 h of incubation, the same pro-oxidant effect was observed even at IC_50_ concentrations (Figure 4B).

Phenolic compounds have been demonstrated to possess antioxidant effects [41], although they may also exhibit pro-oxidant properties. This latter effect has been detected in cancer cells and has been linked to pro-apoptotic action. The dualistic behavior of plant phenolic compounds, exhibiting both antioxidant and pro-oxidant properties, is contingent upon the type of cell, the concentration, the chemical structure, and the pH state [95,96].

In this regard, numerous researchers have indicated that the mechanisms underlying the anti-cancer effects of mushroom extracts may be related with the generation of ROS by the compounds present in the extract, resulting in a pro-oxidant effect on cancer cells [79,97,98]. Trehalose is a disaccharide that can also induce autophagy, which could suppress tumor growth and progression by altering oxidative stress in cancer cells due to changes in mitochondrial potential [62]. Consequently, the phenolic compounds and trehalose present in the extracts of BE may be the cause of their pro-oxidant effect which triggers programmed cell death by apoptosis and autophagy.

Thioredoxin (TrxR) is an enzyme that contains selenocysteine residues and plays a role in maintaining the redox balance within the cell. The inhibition of this enzyme results in a pro-oxidant state within the cell. This enzyme is highly expressed in certain cancers [99]. For this reason and given that BE produces a pro-oxidant effect on Caco-2 cells, it was investigated whether the activity of this enzyme was altered by the action of the extracts. As illustrated in Figure 4C, the BE extracts demonstrated a notable reduction in TrxR activity at an IC_50_ concentration, with a 48 h incubation period. This inhibition may be attributed to alterations in the intrinsic affinity of the enzyme for different chemical forms in which selenium could be found in BE extracts. The observed decrease in TrxR activity is consistent with the pro-oxidant effect previously identified.

TrxR has gained more and more attention in recent times as a significant tumor growth modulator. Thus, the use of TrxR is a promising approach to treating cancer.

### 3.6. Anti-Inflammatory Effect of BE Extracts on Colon Cancer Cells

The cyclooxygenase-inducible isoform, COX-2, is known to be overexpressed in numerous solid tumors [100]. Epidemiological and clinical studies with selective and non-selective COX-2 inhibitors have demonstrated a correlation between COX-2 and cancers such as colorectal, gastric, breast, and prostate [12].

In the context of nitric oxide (NO) signaling in colorectal cancer, it is widely recognized that the overproduction of NO by the inducible nitric oxide synthase (iNOS) triggers inflammation and impacts tumor progression in the colon through diverse pathways. Its pivotal role involves the inhibition of DNA repair enzymes and the prevention of apoptosis via caspase nitrosylation, as previously demonstrated by Hofseth et al. and Rao et al. [101,102]. Thus, iNOS and COX enzymes and the synthesis of prostaglandins may represent new goals for both chemoprevention and anticancer therapy [103]. In this way, extracts from certain mushrooms inhibited inducible iNOS and COX-2 and suppressed NF-kB in RAW2647 cells [104]. Additionally, mushroom extracts blocked the inducible expression of a human *COX-2* and iNOS promoter-driven reporter gene [105].

Phenolic compounds, and flavonoids in particular, have been extensively documented as efficacious inhibitors of catabolic processes due to their antioxidant and anti-inflammatory properties [106]. In addition, trehalose has been shown to inhibit tumor growth and cancer cell proliferation due to its capacity to reduce inflammation in tumor tissue [59].

**Figure 5:**
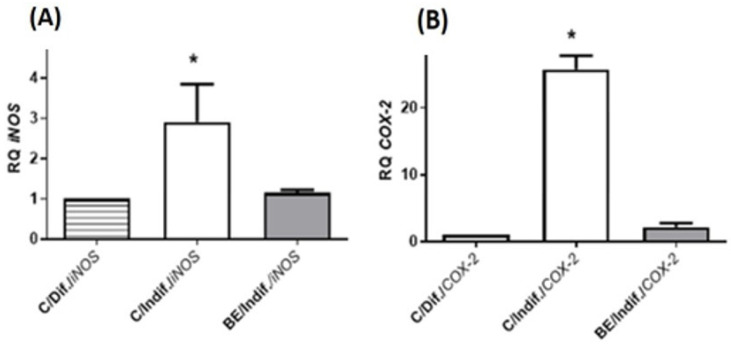
The impact of *B. edulis* extract (BE) at 1500 μg/mL (IC_50_) for 48 h on mRNA levels of *iNOS* (**A**) and *COX2* (**B**) in undifferentiated Caco-2 cells versus their differentiated counterparts. * *p* < 0.05 compared to control (differentiated cells).

The findings of this study reveal that undifferentiated Caco-2 cells display significantly higher gene expressions of *iNOS* and *COX-2*, with levels 2.9 ± 1.8 and 23.8 ± 1.1 times higher, respectively, than their differentiated counterparts. This confirms the pro-inflammatory state of these cells. Treatment with 1500 μg/mL of BE extracts for 48 h resulted in a reduction in the expression of both markers in undifferentiated cells, bringing them closer to the levels observed in differentiated cells (Figure 5). This highlights the potential of BE extracts in mitigating inflammation in colon cancer cells. These data are consistent with those reported by De Felice et al. [107], who observed a reduction in the mRNA expression of *COX-2*, *TNF-a*, and *IL1-β* in non-stimulated Caco-2 cells following exposure to alpha-glucans derived from shiitake mushrooms (*Lentinula edodes*). Similarly, Sergent et al. [108] reported that treating non-stimulated Caco-2 cells with ellagic acid and ferulic acid led to a reduction in genes associated with inflammation and fatty acid metabolism. Along these lines, O’Leary et al. [109] demonstrated that quercetin and its conjugated forms could reduce *COX-2* mRNA expression and/or activity in both unstimulated and IL1-β-stimulated Caco-2 cells.

**Figure 6:**
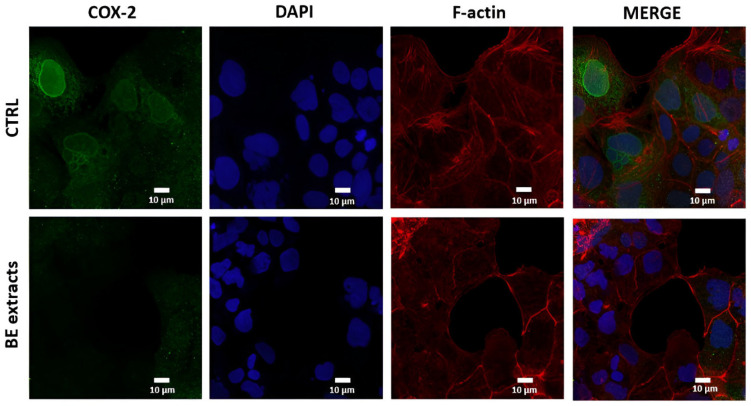
Immunofluorescence assessment of COX-2 in undifferentiated Caco-2 cell line is shown in the upper panel (CTRL), and following treatment with 1500 μg/mL of *B. edulis* (BE) extracts for 48 h in the lower panel. COX-2 staining appears in green, DAPI in blue, and F-actin in red.

Figure 6 depicts the immunofluorescence staining of COX-2, with DAPI and F-actin counterstaining. In the undifferentiated Caco-2 cells (CTRL), the COX-2 expression is marked by increased green fluorescence around the epithelial region, indicating the presence of COX-2-positive cells. This expression notably decreased upon the administration of 1500 μg/mL of BE extract for 48 h. These findings are consistent with those observed at the mRNA level, thus highlighting its potential to hamper colonic inflammation through the reduction in the levels of COX-2 and iNOS inflammatory markers.

### 3.7. Protective Effect of the Extracts on the Intestinal Barrier

Bioactive compounds, present in plants and fungi, play a significant role in the prevention of gastrointestinal diseases related to the excess of free radicals, like cancer, colitis, etc. [110,111]. Taking into account the content of bioactive molecules (Table 3, Table 4 and Table 5) and given that these extracts showed an antioxidant effect determined by FRAP, DPPH, and ABTS (Table 3), it was deemed pertinent to evaluate their effect, at different concentrations and with an incubation time of 24 h, on an intestinal barrier model (differentiated cells) in the presence and absence of an oxidative stress inducer such as H_2_O_2_. Upon reaching confluence, Caco-2 cells undergo a transformation into differentiated cells, acquiring the phenotypic characteristics of normal (non-cancerous) enterocytes [112,113]. Therefore, it has been determined that differentiated Caco-2 cells are a suitable in vitro model of the intestinal barrier.

**Figure 7:**
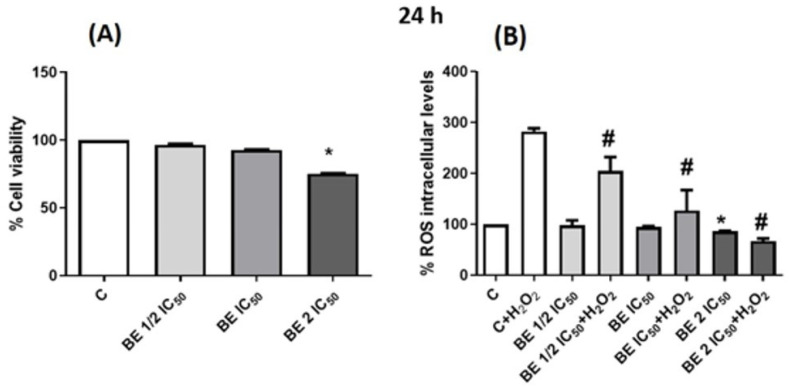
(**A**) Viability of Caco-2 differentiated cells after 24 h incubation with different concentrations (1/2 IC_50_, IC_50_, and 2 IC_50_) of *B. edulis* extracts (BE). (**B**) Measurements of ROS intracellular levels in presence of H_2_O_2_ (80 mM, 20 min) after 24 h incubation with different concentrations (1/2 IC_50_, IC_50_, and 2 IC_50_) of BE extract. * *p* < 0.05 compared to control (without treatment). # *p* < 0.05 compared with control + H_2_O_2_.

The results showed a remarkable antioxidant action due to the prevention of the formation of H_2_O_2_-induced ROS (Figure 7B). The results were adjusted concerning the viability obtained at the different concentrations assayed, observing an effect only at the concentration of 2 IC_50_ (Figure 7A). These results are in line with those found for *Calendula officinalis* extracts with the ability to inhibit the increase in ROS levels derived from the exogenous addition of H_2_O_2_ [114].

Our results obtained for the BE extracts suggest that they could have potential applications in the management of gastrointestinal diseases related to oxidative stress, including cancer and inflammatory bowel diseases (IBDs).

## 4. Conclusions

This study shows that the hydroethanolic extracts of BE contain a large amount of bioactive compounds such as sugars (namely trehalose), phenolic compounds (flavonoids and phenolic acids), and minerals. Among these, selenium and zinc are of particular interest due to their bioactive capacity. The presence of these compounds in the extract suggests that they may possess antioxidant activity, which could indicate their potential as anticancer or chemopreventive agents. The data demonstrate that BE extracts inhibit the proliferation of human colon carcinoma cells (Caco-2) by inducing cell cycle arrest in the G0/G1 stage and inducing cell death by apoptosis and autophagy. Additionally, alterations in the mitochondrial potential and increased caspase-3 activity were observed. The extracts altered the redox balance of the cells, resulting in increased cellular ROS levels and decreased activity of the Trx enzyme. In addition, these extracts demonstrated anti-inflammatory properties by reducing *COX-2* and *iNOS* gene expression and COX-2 protein levels. Conversely, BE extracts demonstrated antioxidant effects on differentiated enterocytes, thereby protecting the intestine from oxidative stress. The results obtained provide a rationale for further studies to determine the effect of these extracts in vivo.

## Figures and Tables

**Table 1:** Chemical composition of dried *B. edulis* (composition out of 100 g).

Composition (%)	
Carbohydrates	58.1 ± 2.8
Extractives	
Water	36.8 ± 0.3
Ethanol	43.5 ± 0.5
Protein	19.7 ± 0.9
Fat	2.4 ± 0.1
Ash	5.4 ± 0.1
Energy (kcal/100 g)	333

**Table 2:** Mineral composition of dried *B. edulis*.

Minerals (mg/kg dry BE)	
K	21,500 ± 471
P	4039 ± 69
Mg	645 ± 20
Na	614 ± 19
Ca	138 ± 8
Zn	106 ± 14
Al	110 ± 6
Fe	66 ± 9
Se	62 ± 7
Mn	17 ± 0.4
Cu	13 ± 0.3
Ba	6.5 ± 0.2
Ni	5.4 ± 0.1

**Table 3:** Total phenolic (TPC) and flavonoid (TFC) content, individual phenolic compounds, and antioxidant activity (FRAP, DPPH, and ABTS) of *B. edulis* extracts.

**TPC** (mg GAE/g dry BE extract)	79 ± 4
**TFC** (mg CE/g dry BE extract)	7.9 ± 0.9
**Individual phenolic compounds** (µg/g dry BE extract)	
Kaempferol	1.4 ± 0.2
Quercetin	99 ± 7
Taxifolin	259 ± 20
Rutin	465 ± 35
2,4-Dihydroxybenzoic acid	53 ± 5
Ferulic acid	85 ± 6
Ellagic acid	532 ± 44
Rosmarinic acid	97 ± 5
Cinnamic acid	172 ± 33
**Antioxidant activity**	
**FRAP (µmol Fe^2+^/g** dry BE extract**)**	103 ± 6
**DPPH (µmol TE/g** dry BE extract**)**	17 ± 0.1
**ABTS (µmol TE/g** dry BE extract**)**	52 ± 2.5

**Table 4:** Free sugar composition of *B. edulis* extracts.

Sugars	Content (mg/g BE Extract)
Trehalose	341 ± 0.1
Rhamnose	51 ± 6
Mannitol	23 ± 0.5
Glucose	16.6 ± 0.3
Fructose	6.7 ± 0.2

**Table 5:** Mineral composition of *B. edulis* extracts.

Minerals **(mg/kg Dry BE Extract)**	
K	27,691.9 ± 156
P	3234.3 ± 112
Na	1416.0 ± 66
Mg	572.4 ± 36
Ca	139.7 ± 13
Zn	49.8 ± 5.1
Se	26.9 ± 1.2
Cu	12.8 ± 1.1
Fe	12.7 ± 2.3
Mn	4.1 ± 0.4
Ni	2.4 ± 0.1
Ba	<LOQ

LOQ: limit of quantification.

**Table 6:** IC_50_ (μg/mL values of BE extracts) of Caco-2 cells at different incubation times with *B. edulis* extracts.

24 h	48 h	72 h
>2000	1880 ± 18	1509 ± 43

## Data Availability

Data is contained within the article or Supplementary Material.

## Supplementary Materials

The following supporting information can be downloaded at: https://www.mdpi.com/article/10.3390/antiox13080908/s1.
