# Supplementary material for: Biological Properties of Boletus edulis Extract on Caco-2 Cells: Antioxidant, Anticancer, and Anti-Inflammatory Effects
Source: Antioxidants (Basel). 2024 Jul 27;13(8):908. doi: 10.3390/antiox13080908 (PMC11352050; doi:10.3390/antiox13080908)
Supplement: Supplementary file 1 [file antioxidants-13-00908-s001.zip › antioxidants-3079060-supplementary.pdf]

Supplementary Table S1: Retention time (RT), wavelength (λ) calibration equation, coefficient of linear correlation (R^2) and purity for the different tested phenolic compounds.

**Compounds***	**RT (min)**	**λ (nm)**	**Calibration equation**	**R^2**	**Purity (%)**
2,4-dihydroxybenzoic acid	3.792	280	Y=(14712.3)X+(-49796.2)	0.9978	≥97
ferulic acid	11.015	320	Y=(16537.2)X+(-136229)	0.9916	≥99
ellagic acid	13.129	250	Y=(16782.3)X+(-77633.8)	0.9912	≥95
cinnamic acid	14.030	280	Y=(23166.2)X+(-61293.7)	0.9998	≥99
rosmaniric acid	12.846	329	Y=(5129.3)X+(-61557.1)	0.9970	≥98
rutin	11.755	350	Y=(3112.7)X+(-1409.97)	0.9999	≥94
taxifolin	14.366	280	Y=(7540.12)X+(-50805.6)	0.9979	≥85
quercetin	13.789	370	Y=(10695.1)X+(-49241.7)	0.9961	≥98
kaempferol	15.089	370	Y=(11146.9)X+(13097.2)	0.9960	≥97

*Obtained from Sigma Aldrich.
